# (In) visibility of notifications of violence against children and adolescents registered in a municipality in southern Brazil

**DOI:** 10.17533/udea.iee.v37n2e11

**Published:** 2019-09-19

**Authors:** Priscila Arruda da Silva, Valéria Lerch Lunardi, Rodrigo Dalke Meucci, Simone Algeri, Michele Peixoto da Silva, Flávia Pivoto Franciscatto

**Affiliations:** 1 Ph.D. Nurse. Federal University of Rio Grande - FURG. Rio Grande, RS- Brazil. Email: patitaarruda@yahoo.com.br Federal University of Rio Grande Rio GrandeRS Brazil patitaarruda@yahoo.com.br; 2 Ph.D. Nurse. Retired professor, Federal University of Rio Grande FURG. Rio Grande, RS - Brazil. Email: vlunardi@terra.com.br Federal University of Rio Grande Rio GrandeRS Brazil vlunardi@terra.com.br; 3 Physiotherapist, Ph.D. Professor, Federal University of Rio Grande FURG. Rio Grande, RS - Brazil. Email: rodrigodalke@yahoo.com.br Federal University of Rio Grande Rio GrandeRS Brazil rodrigodalke@yahoo.com.br; 4 Nurse, Ph.D. Professor at the Federal University of Rio Grande do Sul. Porto Alegre, RS - Brazil. Email: simone.salgeri@gmail.com Federal University of Rio Grande do Sul Porto AlegreRS Brazil simone.salgeri@gmail.com; 5 Social Assistent, Master´s degree. Prefeitura Municipal do Rio Grande, RS - Brazil. Email: chele.p@hotmail.com Prefeitura Municipal do Rio Grande RS Brazil chele.p@hotmail.com; 6 Nurse, Ph.D. Palmeira das Missões, RS - Brazil. Email: flaviapivoto@yahoo.com.br Palmeira das Missões Brazil flaviapivoto@yahoo.com.br

**Keywords:** nursing, domestic violence, child advocacy, mandatory reporting, registries., enfermería, violencia doméstica, defensa del niño, notificación obligatoria, sistema de registros.

## Abstract

**Objective.:**

To know the perception of health, education and social service professionals about the records and notifications of violence against children and adolescents, carried out in a municipality in the south of Brazil.

**Methods.:**

This is an exploratory, descriptive, and qualitative approach, specifically developed in places that integrate children and adolescents victims of violence. Ten professionals participated, including three nurses, one doctor, two social workers, two psychologists, one tutor, and one educator. Data collection was performed through a semi-structured interview. The statements were submitted to discursive textual analysis.

**Results.:**

The analysis showed that the act of recording and reporting violence against children and adolescents is still not a routine practice for health professionals. The registration and formal communication of the information should be considered as a priority; however, the results showed that the protection of the victim seems to overlap with the registry. The study identified important elements in strategies for coping with violence against children and adolescents: centralization of notifications in a single service; creation of a notification flow; existence of an advisory team to deal with cases of violence; and completion of compulsory notification by education and social assistance professionals.

**Conclusion.:**

For the professionals, the routine attendance of situations involving violence, but not formalized through the notification form, has contributed to the underreporting and invisibility of the cases.

## Resumen

**Objetivo.:**

Conocer la percepción de los profesionales de la salud, educación y servicio social acerca de las notificaciones de violencia contra los niños y adolescentes, realizadas en un municipio del sur de Brasil.

**Métodos.:**

Se trata de una investigación exploratoria, descriptiva, cualitativa, desarrollada específicamente en centros que integran niños y adolescentes víctimas de violencia. Participaron en el estudio diez profesionales: tres enfermeros, un médico, dos asistentes sociales, dos psicólogos, un consejero tutelar y un educador. La recolección de los datos se realizó mediante entrevistas semiestruturadas. Los testimonios se sometieron al análisis textual discursivo.

**Resultados.:**

El análisis realizado muestra que el acto de registrar y notificar la violencia contra los niños y adolescentes aún no es una práctica usual en la rutina de los profesionales participantes. El registro y comunicación formal de la información debería ser considerada como prioridad, sin embargo, los resultados apuntan a que la protección de la víctima parece superponerse al registro. El estudio identificó elementos importantes en el direccionamiento de estrategias de enfrentamiento de la violencia contra niños y adolescentes, a saber: centralización de las notificaciones en un único servicio; creación de un flujo de notificaciones; existencia de un equipo asesor para la atención de los casos de violencia; diligenciamiento de la ficha de notificación por los profesionales de educación y asistencia social.

**Conclusión.:**

Para los profesionales la atención rutinaria de situaciones que involucran violencia, pueden no ser formalizadas a través de la ficha de notificación, lo que está contribuyendo en la subnotificación e invisibilidad de los casos.

## Introduction

Violence against children and adolescents is characterized as an epidemic with serious consequences for individual and collective health.(1) According to the results of the study “The Influence of Geographical and Economic factors in estimates of childhood abuse and neglect using childhood *trauma questionnaire: a worldwide meta-regression analysis*, Brazil is the country with the highest estimates of child maltreatment in the world.(2) The complexity of the phenomenon that is usually treated in a veiled way by the aggressors and victims justifies and demands from the health professionals, the notification of cases of violence against children and adolescents by the competent authorities. According to Brazilian law, any violation of the rights of children and adolescents must be notified.

By Ordinance 1271/2014,(3) compulsory notification must be performed in all health units of public or private services. In cases of sexual violence or suicide attempt, after knowing the occurrence, the notification should be done. In other types of violence, it can be carried out weekly.

The cases of violence against children and adolescents are included in the item on domestic violence and/or another violence type, considered as injuries, as they represent damage to the physical or mental integrity of the individuals, as they are caused by harmful circumstances, such as injuries resulting from interpersonal violence, aggression, and maltreatment.(4) These injuries must be reported to the local authority, carried out by health professionals or heads of health institutions, even if it is a suspicion, by completing the notification form, whose data are inserted in the Notification of Injury Information System.(5)

The scientific production about the notification of intra-family violence against children and adolescents has intensified since the 1990s and has been the subject of a study by several national and international authors on the duty to notify it.(6,7) However, despite this obligation, it is understood the plurality that involves violence, necessary to contextualize the problem and discuss it based on the new tendencies of care, which involve community services, and the daily practices of the professionals in the network. A study by Garbin *et al.* (8) identified professionals' difficulties in reporting violence against children and adolescents. These difficulties include the lack of professional training on reporting and actions to be taken, fear of retaliation by the aggressor, structural issues related to the unsatisfactory performance of the competent bodies in complying with protective measures, difficulties or constraints in completing the notification form.

Knowing the perception of professionals who work directly in places of entry and hospitalization of children and adolescents victims of violence, can point out possible ways to overcome gaps and deficiencies faced by these professionals, giving visibility to a phenomenon that is still veiled in society. Thus, this study aims to know the perception of professionals, about the records and reports of violence against children and adolescents, carried out in the social and health services of a municipality in the South of Brazil.

## Methods

This is an exploratory, descriptive, and qualitative approach, developed in a municipality in the extreme south of Brazil, specifically in places that integrate children and adolescents victims of violence. Ten professionals in which three were nurses, one was a lawyer, one was a doctor, one was a social worker, two were psychologists, one was a tutor, and one was an educator participated in the study. These professionals work in the following services: Family Health Strategy, Family Health Support Center, Specialized Referral Center in Social Assistance, Tutelary Council, Secretariat of Citizenship and Social Assistance, Health and Teaching Secretariat. The following inclusion criteria were used: to know the work of the municipality on the theme. The time of service was also considered, with preference to professionals who had been working in the workplace for over a year. The exclusion criterion was the non-location of the participant, after three attempts to meet.

The ethical prerogatives of Resolution 466/12 of the National Health Council(9) were respected and the study was approved by the Health Research Ethics Committee of the Federal University of Rio Grande under CAAE: 49775415.8.0000.5324. When signing the Free and Informed Consent Form, the interviewees were explained the purpose of the research and guaranteed the right not to participate or to interrupt their participation at any time. Data collection took place through a semi-structured interview, from April to June 2016, focusing on the existence of standardized instruments for reporting, record quality and continuous flows of information for its accomplishment. Besides the semi-structured interview, consultations were used in the forms of notification of aggravated violence, and documents were available by the coordinators of the Secretariats of Health, Education and Social Assistance.

The study was carried out in the work environment of each professional, individually, at a time and place previously scheduled, saving the adequate progress of the professionals' work. Some care was taken to create a private and safe environment: an explanation of the purpose and objectives of the study, ethical issues related to human research, such as the right to refuse to participate in research, respect for anonymity through code identification that guarantees the confidentiality of the information obtained, through Letter E, following the order of interviews. The content of the interviews was subjected to a discursive textual analysis, following the rigorous and thorough reading, and its deconstruction, highlighting the units of analysis. (10)

## Results

Three categories emerged in the process of data analysis: (De) valorization of notification and records of violence against children and adolescents; (In) visibility of information records on violence and Municipal performance in coping with violence: notification as a guarantee of rights

### Characterization of study individuals

**Table 1:** Sample of individuals interviewed, according informers and institution

Informers	Institution/services
E1	Health
E2	Social
E3	Health
E4	Health
E5	Social
E6	Education
E7	Health
E8	Health
E9	Social
E10	Social

Regarding the characteristics of the participants of this study, nine were female and one male, aged between forty-one and fifty-eight. Working time in the institutions ranged from two to twelve years.

(De) valorization of notification and records of violence against children and adolescents

According to the report of the professionals, despite their obligation and their value as a way to guarantee the rights of children and adolescents in their care, notification is a practice still little used in the routine of professionals, perhaps due to the absence of an organization institutional, as evidenced here*: we know that if we suspect, we must notify it, but in some cases of violence we see, the professional does not do it, it is solved in the unit with the family, perhaps because they [professionals] understand that to notify responsible bodies, to the Tutelary Council, sometimes it is not resolving (E1); Depending on the type of violence, we did not make formal notification for lack of information, but for fear of losing the family (E3). I think the most important thing is the protection of this child (E7). It happens a lot that we make the notification to the responsible organs and nothing happens. Besides not being resolved, it still exposes the professional (E5). Because it is a complex issue, this has not been an easy task for professionals, given the fear of the repercussions that violence has generated for both families and working professionals. Talking in a notification is easy, I know its importance, but many colleagues had to change units because they were threatened (E1). We have a professional only to fill the notifications so you cannot identify who notified it (E4). In situations of violence, I believe that the key would be a team that could support us, even though the Family Health Support Center has helped us a lot (E3). However, at the same time that the professionals recognize a certain devaluation of the notification, they identify that the municipal management has invested in training on the individual and emphasize an intense debate about the completion of the notification form.*

### (In) visibility of information records on violence

Regarding the information obtained, through the medical records of different investigated services, the researcher can verify the lack of details in the collected materials, which has hindered to elucidate many cases of violence. There was incomplete data in the records of the referral services, in the records of the Notification of Injury Information System, *Disque Denuncia* 100, not including the necessary information. Another relevant fact in this study is the notifying body. The notifications that arrive until the specialized service are through denunciations made by dialing 100, not having the information of the professional who has notified. According to the professionals' reports, the fragmentation of records has led to deficiencies, mainly in diagnosis and notification by professionals and institutions, besides apparently constituting an act disjointed with the existing protection network in the municipality. According to Education, Health and Social Assistance professionals: *The accuracy of the problem of violence that the municipality faces is not known, each secretariat [health, education, social assistance] has its instrument for registration (E2). The notifications should be centered on a single service, but in practice, this does not happen (E8). There have already been targeted meetings for this purpose, but they are still on paper (E10). In education, teachers notify the coordination, we investigate the situation and pass the case to the tutelary board, but nothing formal (E6).*

According to one respondent's report, there have been several attempts to standardize the instruments used in reporting units. In the units of the respective secretariats, (health, social assistance, and education), training was carried out to mobilize professionals to incorporate the notification into their routine work, but without success. Another important device is to enter the Notification of Injury Information System, noticing that in the municipality, apparently, there are no cases of violence registered until then, but we know that at least one case of violence per day is accompanied by protection services. Therefore, the communication actions have required that demand and promote continuous flows of information to fulfill its objectives, especially with the protection organs. However, the Tutelary Council, as a body for the protection and management of information on violation of rights, does not have a system that allows the visualization of cases of violence that reaches the service, which further weakens the identification of a violation of rights.

### Municipal action in coping with violence: notification as a guarantee of rights to children and adolescents

The results showed that in the municipality, there is a lack of municipal management investments in the consolidation of public policies directed at children and adolescents, especially regarding notification. According to representatives of the Secretariat of Citizenship and Social Assistance, there is no commission to confront sexual violence against children and youth in the municipality. Thus, the conditions offered by the municipal manager to the protection services observed and pointed out by the service coordinators are a problem that persists for years. Violence is linked to a social context permeated by barriers that hinder the notification process. *The municipality lacks a service policy, such as a structure capable of providing protection services with conditions to perform effective work, with computers available for reference and against reference (E8), adequate physical conditions (E9), systems that provide an accurate diagnosis of the situation of violence in the municipality and that allows data to be crossed between the involved organs (E10).*

## Discussion

Although professionals advocate the protection of children and adolescents, they do not register a case of violence, bringing up a problem present in every day that is underreporting, which can corroborate so many cases are excluded from official estimates. (11-13) Underreporting of violence is still a reality, not only in the researched context, but in many countries, perhaps because it is culturally recognized as a punishment process, and not as assistance, impairing the true dimension of violent events.(13) According to a report by the European Union Agency for Fundamental Rights, almost all of the notifications in the European Union are compulsory. However, not all countries have institutional notification protocols or documents that establish the responsibilities of professionals. Also, another problem identified in countries such as Denmark and Lithuania and strongly related to underreporting is the anonymity of professionals, which has discouraged professionals from reporting.

In Brazil, little is known about the standards adopted for its effective operationalization. Each state has its notification flow, little is known about the mobilization of resources effectively triggered by compulsory notification by health professionals.(15) Although there is no institutional culture to report a situation of violence, this act is essential in confronting violence against children and adolescents and in the process of restoring their rights. Besides stopping the abuse and initiating measures of protection and assistance to children and adolescents in situations of violence and their families, it also provides information for assessing the local situation and the need for public investments.(16)

The lack of detailed information in the medical records, incorrect or incomplete data are problems pointed out in other studies,(12,15) which has hindered to confirm or elucidate cases of violence. Possibly, because of the lack of knowledge of the professionals about the support network for victims, disbelief in their efficiency or fear of the professional being compromised, professionals have stopped making the notification.(17) Considering that information is essential for knowledge and for decision making, the results demonstrate an apparent lack of an institutional culture of valuation of records. The information that should be considered as a priority, because of its importance, according to the results presented, the protection of the victim seems to override the registry. It is clear that statistics on violence against children and adolescents reveal only part of the cases of violence that children and adolescents have subjected daily.(18) Facing this fact, it is evident that there is a need to improve information systems, to improve its quality, integrating other systems such as those managed by the Public Ministry and judiciary.

There are many challenges in the area of health that affect the qualification and support of professionals in situations of violence. Professionals need to feel secure and supported to make the notification and to visualize the results of their action. Although the notification may not have a transforming effect on immediate reality, it is certainly an instrument that allows visibility to the manifestations of violence, sizing the intensity and characteristics of the phenomenon. (18)

## Conclusions

The study identified important elements for coping strategies on violence against children and adolescents: centralization of notifications in a single service; creation of a notification flow; the existence of an advisory team to deal with cases of violence; and the completion of compulsory notification by education and social assistance professionals.

The adoption of standardization in information is a demand pointed out by professionals since 2002 when the referral service specialized in social assistance in the municipality was implemented. The flow of notifications seems to be an urgent need pointed out by the interviewees, given the small number of data on violence. Thus, the adoption of a compulsory notification form in all secretariats has been working (health, education and social assistance), so the notifications are centered on epidemiological surveillance, which is responsible for registering in the Notification of Injury Information System. The existence of an advisory team to deal with cases of violence could be characterized as an institutional policy to address this problem. This would allow the involvement of all professionals, so they can deal, investigate, diagnose, attend and refer cases with institutional support, and not act by developing only individualized and fragmented actions.

In the reality found in the municipality, it is relevant to approach the theme, so all areas of knowledge, working with children and adolescents, can exercise their co-participation, giving visibility to a problem as serious as violence. This research shows important contributions to collective health, considering that, to ensure the protection and guarantee of the rights of children and adolescents and overcoming situations that violate their rights, requires knowledge in the municipalities are articulating in the face of cases of violence. Thus, the study highlights the need for a greater commitment in the preparation of professionals for the phenomenon, since the notification has been an increasing challenge for professionals in their different areas of activity, which has been confronted with difficulties arising from this training. It is also suggested new studies that will complement knowledge gaps and contribute to improving the quality of life of the child population.

As a limitation of the study, there is the accomplishment in a single scenario, since the actions of notification of violence and sexual exploitation against children and adolescents have a national scope. The replication of the study in other realities may increase the visibility of the obstacles to the necessary implementation of the notification of violence against children and adolescents in health services.

## References

[1] Covell K, Becker JO (2011). NGO Advisory Council for Follow-up to the UN Study on Violence against Children. Five years on: a global update on violence against children.

[2] Viola TW, Salum GA, Kluwe-Schiavon B, Sanvicente-Vieira B, Levandowsky ML, Grassi-Oliveira R (2016). The influence of geographical and economic factors in estimates of childhood abuse and neglect using the Childhood Trauma Questionnaire: A worldwide meta-regression analysis. Child Abuse Neglect..

[3] Brasil. Ministério da Saúde (2014). Portaria nº 1271, de 6 de junho de 2014. Define a Lista Nacional de Notificação Compulsória de doenças, agravos e eventos de saúde pública nos serviços de saúde públicos e privados em todo o território nacional, nos termos do anexo, e dá outras providências. Diário Oficial da União.

[4] Egry EY, Apostolico MR, Morais TCP. (2018). Notificação da violência infantil, fluxos de atenção e processo de trabalho dos profissionais da Atenção Primária em Saúde.. Cien. Saude Colet..

[5] Cezar PK, Arpini DM, Goetz ER. (2017). Registros de Notificação Compulsória de Violência Envolvendo Crianças e Adolescentes.. Psicol. Cien. Prof..

[6] Delziovo CR, Bolsoni CC, Coelho EBS. (2018). Quality of records on sexual violence against women in the Information System for Notifiable Diseases (Sinan) in Santa Catarina, Brazil, 2008-2013.. Epidemiol. Serv. Saude..

[7] Nunes AJ, Sales MCV. (2016). Violence against children in Brazilian scenery.. Cien. Saude Colet..

[8] Garbin CAS, Dias IA, Rovida TAS, Garbin AJI. (2015). Desafios do profissional de saúde na notificação da violência: obrigatoriedade, efetivação e encaminhamento.. Cien. Saude Colet..

[9] Brasil. Ministério da Saúde Conselho Nacional de Saúde (2012). Resolução nº 466, de 12 de dezembro de 2012. Aprova as diretrizes e normas regulamentadoras de pesquisa envolvendo seres humanos.

[10] Moraes R, Galiazzi MC. (2011). Análise textual discursiva.

[11] Rolim ACA, Moreira GAR, Corrêa CRS, Vieira LJES. (2014). Subnotificação de maus-tratos em crianças e adolescentes na Atenção Básica e análise de fatores associados.. Saúde Debate..

[12] Waiselfisz JJ. (2012). Mapa da violência. Crianças e Adolescentes do Brasil.

[13] Moreira GAR, Vieira LJES, Deslandes SF, Pordeus MAJ, Gama IS, Brilhante AVM. (2014). Fatores associados à notificação de maus-tratos em crianças e adolescentes na atenção básica.. Cien. Saude Colet..

[14] European Union Agency for Fundamental Rights (2015). Provisions on professionals’ legal obligation to report cases of child abuse, neglect and violence.

[15] Lima JS, Deslandes SF (2011). A notificação compulsória do abuso sexual contra crianças e adolescentes: uma comparação entre os dispositivos americanos e brasileiros.. Interface (Botucatu)..

[16] Deslandes S, Mendes CHF, Lima JS, Campos DS. (2011). Indicadores das ações municipais para a notificação e o registro de casos de violência intrafamiliar e exploração sexual de crianças e adolescentes.. Cad. Saude Publica..

[17] Abath MB, Lima MLLT, Lima PS, Silva MCM, Lima MLC. (2014). Avaliação da completitude, da consistência e da duplicidade de registros de violências do Sinan em Recife, Pernambuco, 2009-2012.. Epidemiol. Serv. Saude..

[18] Santos TM, Cardoso DM, Pitangui AC, Paiva SM, Melo JP, Silva LM. (2016). Completeness of notifications of violence perpetrated against adolescents in the State of Pernambuco, Brazil.. Cien. Saude Colet..

